# The Efficacy of Early Rehabilitation Combined with Virtual Reality Training in Patients with First-Time Acute Stroke: A Randomized Controlled Trial

**DOI:** 10.3390/life14070847

**Published:** 2024-07-05

**Authors:** Ta-Chung Chao, Chia-Huei Lin, Meei-Shyuan Lee, Cheng-Chiang Chang, Chia-Ying Lai, Chien-Yao Huang, Wen-Yuan Chang, Shang-Lin Chiang

**Affiliations:** 1 Department of Physical Medicine and Rehabilitation, Tri-Service General Hospital, School of Medicine, National Defense Medical Center, Taipei 114, Taiwan; ericapple3000@yahoo.com.tw (T.-C.C.);; 2 School of Nursing, National Defense Medical Center, Taipei 114, Taiwan; andyy520@mail.ndmctsgh.edu.tw; 3 School of Public Health, National Defense Medical Center, Taipei 114, Taiwan

**Keywords:** early rehabilitation, virtual reality, stroke, depression, randomized controlled trial, recovery of function

## Abstract

Early rehabilitation has beneficial impacts on functional outcomes for patients with acute stroke. However, whether the addition of virtual reality (VR) training could further improve these patients’ muscle strength, functional recovery, and psychological health is unknown. A randomized controlled trial was conducted on 33 patients with first-time acute ischemic stroke. The patients were randomly assigned using a 1:1 randomization ratio to either the experimental group (EG) or the comparison group (CG). Both groups received early rehabilitation, and the EG received extra VR training during their stay in the hospital. Muscle strength, functional status, and psychological health were assessed before the intervention and at discharge. Generalized estimating equations were used to examine the intervention effects via the interaction of time and group. After adjusting for potential covariates, the EG showed a more significant decrease in depression at discharge than the CG (*ß* = 3.77, *p* = 0.011). There were no differences in muscle strength and functional recovery between groups after intervention. Adding VR training into early rehabilitation facilitates substantial positive effects on psychological health, specifically depression, but not muscle strength and functional recovery, compared to receiving early rehabilitation alone in patients with first-time acute stroke during their hospitalized period.

## 1. Introduction

Ischemic stroke, a condition that accounts for nearly 80% of all stroke cases, is not just a health concern but a pressing societal challenge, particularly in Taiwan [1,2]. The aftermath of a stroke often leaves patients with a significantly reduced level of function, making it arduous for them to resume work. This not only affects the individual’s quality of life but also poses substantial financial challenges for stroke survivors and their families, underscoring the profound societal impact of this health issue [2,3]. As healthcare professionals and researchers, we must address this issue with utmost urgency and importance.

Stroke patients are at a heightened risk of psychological distress, including depression and anxiety. The prevalence of post-stroke depression and post-stroke anxiety is about 30% and 20%, respectively [4]. The patient’s level of disability and dependence significantly contribute to the burden placed on caregivers and the psychological distress experienced by stroke patients [3,5]. Therefore, early rehabilitation that enhances muscle strength and functional recovery may improve psychological health and alleviate the caregiver’s burden. In addition, the guidelines from the American Heart Association/American Stroke Association advocate for early detection and treatment of post-stroke depression [6]. An optimal early intervention model that combines psychological improvement, reduction of depression and anxiety, and enhancement of muscle strength and functional recovery is a beacon of hope for stroke patients.

Rehabilitation in the early phase of acute stroke, typically within the first two weeks, has been proven to positively impact patients’ mobility [7,8,9]. Research has shown that sedentary behavior during this phase is linked to poor functional recovery three months later [10]. While research investigating the effects of early rehabilitation on functional recovery and psychological well-being during hospitalization remains limited, innovative approaches have promising potential. Integrating recreational activities and motivational strategies into rehabilitation programs can enhance patients’ rehabilitation participation, leading to improved functional recovery outcomes [11]. Therefore, early rehabilitation combined with training in recreational activities could be an effective strategy for stroke care.

Virtual reality (VR) training is an increasingly popular recreational activity for stroke patients. VR may facilitate learning and provide a safer training environment than traditional instruction-based training [12], particularly for patients with chronic stroke (6 months or more post-stroke) [13]. Studies have shown that VR interventions can improve cognition, activities of daily living, functional task performance, gait, balance, and overall motor function in individuals with chronic stroke [14,15]. However, other research studies did not observe substantial improvements in functional recovery post-stroke using alternative virtual reality methods [16], highlighting the variability of outcomes in this field. While previous studies have predominantly focused on chronic stroke patients, there is a growing interest in exploring its application in acute stroke settings [17,18]. In 2020, Lin et al. found that a post-stroke program incorporating early rehabilitation and VR training resulted in better recovery at discharge compared to early rehabilitation alone, especially in mood and muscle strength status [19]. These findings underscore the urgent need for more well-designed research on applying VR training in acute stroke settings, which could potentially revolutionize early stroke rehabilitation.

Studies have also demonstrated the potential of VR in stroke patients with innovative approaches. Lanzoni et al. introduced a methodology that offers a more immersive and interactive environment for patients to engage with during their recovery process, highlighting the efficacy of VR in cognitive rehabilitation through tailored serious games [20]. Castillo et al. emphasized the use of exergames for upper-limb rehabilitation, focusing on iterative design and usability analysis [21]. These studies underscore the versatility and potential of VR in stroke rehabilitation but primarily concentrate on chronic stroke patients or single aspects.

The goal of our study is to confirm the efficacy and feasibility of early rehabilitation combined with virtual reality training on muscle strength, functional recovery, and psychological health in patients with first-time acute stroke during the acute phase of stroke recovery while in the hospital. By examining the impact of innovative rehabilitation strategies on physical and psychological outcomes, we aim to contribute to the development of more effective and comprehensive stroke care during hospital stays.

## 2. Materials and Methods

### 2.1. Study Design

We conducted a randomized controlled trial in patients with first-time acute stroke to investigate the effectiveness of early rehabilitation combined with virtual reality training on muscle strength, functional recovery, and psychological health. Participants were divided into two groups: the experimental group (EG) received early rehabilitation and VR training, and the comparison group (CG) received only early rehabilitation. The Consolidated Standards of Reporting Trials (CONSORT) statement was used to report this trial [22].

### 2.2. Participants

Between April 2017 and August 2018, a research assistant recruited participants from a medical center in northern Taiwan. Those who agreed to participate underwent screening by the neurologist and rehabilitation physician. The inclusion criteria included (1) first-time acute ischemic stroke; (2) admission to the hospital within three days of stroke onset; (3) age between 20 and 80 years; (4) ability to communicate with verbal or nonverbal methods and understand Mandarin; (5) had a disability ranging from minimal to moderately severe, evaluated as 1–4 scores by the modified Rankin Scale (mRS) [23]; and (6) agreement to be randomized. Exclusion criteria for the study included (1) diagnosis of global aphasia, transient ischemic attack, visual or auditory impairment; (2) mRS ≥ 5 (severe disability: bedridden, incontinent, and requiring constant nursing care and attention); (3) a history of end-stage renal disease with dialysis, dementia, mental health disorders (particularly major depression), based on both medical records and assessments from the neurologist; (4) patients transferred from other wards, (5) being unable to participate due to other comorbid neurological and musculoskeletal conditions that produce moderate-to-severe physical disability; and (6) prolonged stay in hospital for over three weeks due to other medical diseases (e.g., myocardial infarction, septic shock, cancer) after admission, or length of stay in hospital less than one week due to a decline to treatment and transferred to another hospital for further confirmation of diagnosis and other complementary and alternative therapies.

### 2.3. Study Cohorts and Interventions

Eligible patients were randomly assigned to the EG or CG in a 1:1 ratio and placed in separate rooms. Randomization was conducted using sealed opaque envelopes opened by a research nurse. All participants received standard stroke care and early rehabilitation as conventional therapy. The early rehabilitation program, which consisted of 60 min per session, 5 times per week, was prescribed by a rehabilitation physician and carried out by physical, occupational, and speech therapists, starting 3 to 6 days after admission. The EG received supervised VR training, which was conducted for 5 additional days during the hospitalization in addition to the standard early rehabilitation program, using a wireless Kinect sensor (Microsoft Corporation, Redmond, WA, USA) in a discrete space within the neurological care ward. A separate blinded researcher collected outcome measures to ensure the reliability and validity of the data.

The VR training was conducted by two experienced stroke care nurses/researchers with over 20 years of experience in neurological patient care. The training followed a protocol developed by the research team as in the previous randomized controlled study [19]. Each patient in the EG received individual supervised VR training in a dedicated room. The VR device captured full-body images of the patient, projected them onto a monitor in real time, and allowed them to immerse themselves in virtual environments and interact with virtual objects. The VR training sessions included range of motion, coordination, limb strengthening, trunk stabilization, balance, and cognition training. The detailed content of VR training was reported previously [19]. The VR training sessions were tailored to each participant’s functional abilities and progression throughout the intervention. This customization involved adjusting parameters such as difficulty levels, amplitude, speed, frequency, complexity, and provision of hints during each session.

The Institutional Review Board of Tri-Service General Hospital approved this study (TSGHIRB No. 1-106-05-041, approved on 12 April 2017). This study was retrospectively registered on 3 July 2023 on the ClinicalTrials.gov website (registration number: NCT05929742). This study complied with the principles outlined in the Declaration of Helsinki, and this manuscript adheres to the applicable CONSORT guidelines [22]. All participants were invited to join the study after providing written informed consent. They were assured that their participation was entirely voluntary and agreed to the publication of the data collected.

### 2.4. Measures

Outcome data, such as muscle strength, functional recovery, and psychological health, were gathered at baseline (within 4 h after admission) and discharge (7–21 days later) by a separate researcher blinded to the patient’s treatment conditions to ensure unbiased data collection.

#### 2.4.1. Muscle Strength

Muscle strength was assessed using the Medical Research Council Manual Muscle Testing scale, known for its reliability and validation [24]. This scale is easy to perform, requires no specialized equipment, and involves assessing the patient’s upper and lower limb/extremities muscles against the examiner’s resistance and grading the patient’s strength on a 0–5 scale.

#### 2.4.2. Functional Recovery

Functional recovery was assessed using three well-validated clinical assessment tools: the Postural Assessment Scale for Stroke (PASS), the Functional Independence Measure (FIM), and the Barthel scale. These tools are widely used in real-world settings. The PASS, consisting of two sections with a 4-point scale and a total score ranging from 0–36, is used to assess postural control after stroke [25]. The FIM consists of 18 daily activity items with a total score ranging from 18 to 126. Each item is rated on a 7-point scale ranging from 1 (completely dependent) to 7 (independent), according to the level of independence [26]. The Barthel scale, on the other hand, is a widely used tool to assess individuals’ performance in activities of daily living [27]. Patients with higher scores are more independent than those with lower scores in their daily activities.

#### 2.4.3. Psychological Health

The psychological health of the participants was assessed using the highly valid and reliable Hospital Anxiety and Depression Scale (HADS). This tool, with its 14 items (7 items related to anxiety [HADS-A] and 7 related to depression [HADS-D]), provides a comprehensive and accurate assessment of psychological health [28]. The HADS scores each item from 0 to 3, with a range of 0 to 21 for both depression and anxiety, where higher scores reflect higher levels of depression or anxiety [29].

### 2.5. Data Analysis

The continuous descriptive data are expressed as means with standard deviation (SD), and the categorical data are expressed as numbers with percentages (%). Paired *t*-tests were utilized to compare pre- and post-results, and Student’s *t*-tests and chi-squared tests were used to compare pre- and post-intervention differences between groups. Generalized estimating equations (GEE) were used to estimate the intervention effects of the two groups through a significant interaction of group and time (group × time) [30]. When evaluating the impact of the intervention on muscle strength, functional recovery, and psychological health in this study, the models were adjusted for sociodemographic covariates, length of stay, and comorbidities (i.e., history of hypertension, type 2 diabetes, heart disease, hyperlipidemia, and metabolic syndrome). All statistical analyses were two-tailed, and statistical significance was considered for *p*-value < 0.05.

## 3. Results

### 3.1. Baseline Characteristics of Participants

**Figure 1:**
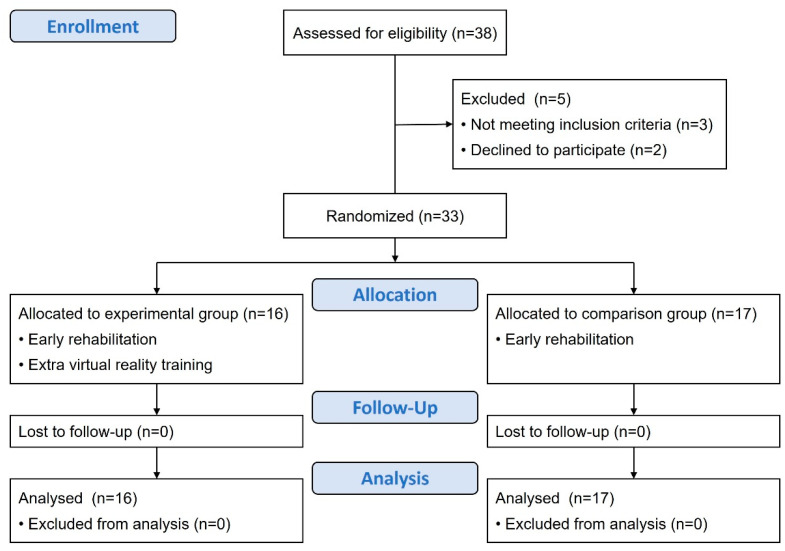
CONSORT flow diagram for participant enrollment, allocation, follow-up, and analysis.

Thirty-eight patients were initially approached. Two participants declined to participate, and three patients did not meet the inclusion criteria (Figure 1). Of the remaining 33 participants with first-time acute stroke, all completed the data collection. There were no significant between-group differences in sociodemographics, comorbidities, and stroke characteristics (Table 1).

### 3.2. Outcome Evaluation

#### 3.2.1. Muscle Strength

There were no significant between-group differences at baseline in the muscle strength of affected and unaffected extremities (Table 2). As shown in Table 2, patients in the EG and CG significantly increased the lower-limb muscle strength of the affected side, with the EG also revealing increased muscle strength in the upper limbs of the unaffected side at discharge. As shown in Table 3, after adjusting for potential covariates, no significant muscle strength increase was observed in the EG compared to the CG.

#### 3.2.2. Functional Recovery

There were no significant between-group differences in functional status at baseline (Table 2). In addition, the CG showed significant improvement in the mRS at discharge compared to baseline (Table 2). After adjusting for sociodemographic and comorbidity characteristics, the GEE analysis showed no significant group × time interactions for functional recovery between EG and CG in the change scores from baseline (Table 3).

#### 3.2.3. Psychological Health

At baseline, the EG reported higher depression scores (12.1 vs. 8.5, *p* = 0.022) compared to the CG (Table 2). At discharge, a significant difference in depression (*p* < 0.001) was observed within the EG (Table 2). As shown in Table 3, participants in the EG reported a significant decrease in depression (*ß* = −2.88, *p* < 0.001) after the intervention. After adjusting for potential covariates, the group × time interaction for depression revealed that the EG had a more significant decrease in the depression status at discharge than the CG (*ß* = 3.77, *p* = 0.011) (Table 3).

## 4. Discussion

Our study assessed the effectiveness of incorporating VR training into early rehabilitation for individuals experiencing their first acute stroke. We found that it enhances muscle strength in the affected-side lower limb, unaffected-side upper limb, and psychological health but does not improve function recovery. After adjusting for potential covariates, VR training only improves psychological health (especially depression state) in patients with first-time acute stroke, with no benefits regarding muscle strength and functional level. These findings emphasize the clinical benefits of VR training when provided alongside early rehabilitation during hospitalization, as it significantly impacts psychological health and can potentially aid in managing depression in this population.

The mental well-being of stroke patients is strongly linked to their level of impairment and reliance on others. Therefore, it is crucial to screen for and treat depression early in the rehabilitation process to improve the patient’s physical outcome and reduce the burden on stroke survivors [3,5]. Our study proves that combining VR training with early rehabilitation can positively impact psychological health, mainly by decreasing depression. While it is unclear whether this effect is due to VR’s recreational or competitive nature [7,11,31], all participants in the experimental group who completed the program suggested that it is feasible and acceptable. Given its potential benefits, VR training could be a valuable complementary approach to conventional post-stroke depression interventions [32]. By improving psychological health and reducing depression, VR training can enhance patient engagement and motivation in rehabilitation, potentially leading to better outcomes [33,34]. Additionally, addressing depression may alleviate the emotional and psychological burden on caregivers and family members, contributing to a more supportive rehabilitation environment.

The positive impact of VR training on upper limbs is well-documented, although the exact mechanism responsible for this effect remains unclear [13]. It may be that repeated task-oriented training, which involves a dynamic stepwise adjustment of the difficulty level and safe simulation of real-world activities of daily living, can enhance neuroplasticity [35]. Meng et al. conducted a systematic review and meta-analysis to assess the effectiveness of transcranial direct current stimulation and VR training for upper extremity rehabilitation. The review included randomized controlled trials that demonstrated the positive impact of frequent and regular VR training on upper-limb function [36]. However, our study demonstrated that after adjusting for potential covariates, the experimental group’s muscle strength improvements became insignificant. In contrast, our previous study showed improved upper limb muscle strength at discharge [19]. Other studies also found that VR training plus conventional therapy was associated with improved upper extremity function with longer or more sessions [37,38]. Our study’s lack of a beneficial effect may be due to the limited intervention times and the shorter follow-up evaluation period. Hence, a more intense, longer, or more frequent treatment may be necessary to create a more considerable influence on first-time acute stroke. The observed initial improvements indicate that the initial findings might have been influenced by external factors not directly related to the VR training. Future studies should include comprehensive baseline assessments and consider a wider range of potential covariates to isolate the effects of VR training on muscle strength accurately.

The potential benefits of VR training in stroke rehabilitation have been widely recognized, yet the evidence supporting its efficacy in functional recovery is still inconclusive [12,13]. While recent systematic reviews and meta-analyses suggest that VR-based rehabilitation programs can improve functional outcomes, particularly in the long term, these studies do not reveal a significant difference compared to standard therapy in the early stage of stroke recovery [17,39]. Our study also found no significant difference between VR and control groups regarding functional recovery during hospitalization and at discharge. The lack of significant findings may be due to the minimal impact of first-time acute stroke on functional status, making it challenging to observe the effects of VR training. Therefore, more trials are required to confirm the effectiveness of early VR training in acute stroke and to determine the optimal treatment timing, frequency, and duration for maximizing its benefits on functional recovery.

Our study showed that adding VR training to early rehabilitation did not improve muscle strength in the lower limb compared to those who received early rehabilitation alone, suggesting an issue either with the initial assessment or that the VR training was not adequately designed to target muscle strength specifically. As lower-limb strength is strongly associated with functional status [40], this lack of improvement in muscle strength may result in no discernible differences in functional status between the groups [31]. Additionally, it is worth noting that most participants could not walk independently during their hospital stay, and the VR training was performed in a seated position. Other studies have found that a more targeted approach focusing on lower-limb resistance training or joint movements may significantly improve muscle strength and functional outcomes [41,42]. Further research is needed to determine whether a more targeted training approach focusing on lower-limb exercises, such as in standing positions or more sessions of kick soccer games, may result in more significant improvements in muscle strength and functional outcomes in acute stroke populations. Furthermore, the VR training program should be rigorously designed to ensure it is tailored to the specific rehabilitation needs of stroke patients, possibly including more dynamic and functional tasks that mimic daily activities.

In addition to the design and execution of VR training programs, ethical considerations are important. VR training must be accessible to a diverse population of stroke patients, including those with varying technological literacy, physical capability, and socio-economic backgrounds. For broad implementation and recovery, VR training must be inclusive and adaptable [43]. Providing equitable access to innovative treatments involves developing user-friendly interfaces, training patients and caregivers on how to use the technology, and ensuring that the cost of VR equipment and training is affordable or covered by insurance [44,45]. Addressing these barriers is crucial for making innovative treatments like VR widely accessible and equitable, ultimately enhancing the overall rehabilitation process for all stroke patients.

The feasibility and acceptability of VR training are highlighted by the fact that all patients in the experimental group completed the program. However, it is important to acknowledge the technological challenges associated with VR training. Specialized equipment, such as sensors, is required, which can be costly and may only be readily available in some healthcare settings [46]. Potential technical issues, such as software glitches or hardware malfunctions, can disrupt training sessions and affect the continuity of care [47]. Additionally, patients may have varying levels of comfort and familiarity with technology, and healthcare providers need adequate training to integrate VR into rehabilitation protocols effectively [35]. Future studies should explore these technological challenges in more detail and develop strategies to address them, ensuring that VR training is feasible and acceptable for widespread use in stroke rehabilitation.

Although our study contributes to the growing body of evidence on the effectiveness of VR training in stroke rehabilitation, several limitations should be considered. Firstly, the sample size was relatively small, which may limit the generalizability of our findings. The effectiveness of VR training may vary across different types of stroke patients, including those with recurrent strokes, chronic stroke survivors, or those with varying levels of residual impairment. Secondly, we did not conduct long-term follow-up evaluations, which makes it difficult to determine the sustained effects of VR training on functional recovery. This limitation could lead to underestimating the intervention’s benefits and potential side effects. Thirdly, this study was conducted in a single urban medical center, which may limit the generalizability of the findings to other regions or cultural groups. Given these limitations, caution should be exercised when interpreting the results of our study. We recommend that future studies consider combining early rehabilitation with VR training, especially for stroke patients with depression and anxiety, which may improve their overall rehabilitation outcomes. Further research with larger sample sizes, extended follow-up periods, and diverse stroke populations is warranted to assess the durability of VR training benefits, identify long-term issues, determine if similar psychological benefits can be achieved, and whether functional outcomes can be improved.

## 5. Conclusions

Early rehabilitation combined with VR training demonstrates significant benefits in improving psychological health, particularly by reducing depression levels, but not muscle strength and functional recovery, compared to receiving early rehabilitation alone in patients with first-time acute stroke during their hospitalized period. The statistical analysis showed that the decrease in depression levels was statistically significant after adjusting for potential covariates, indicating a strong effect of VR training on mood improvement.

Future research should explore more intensive and prolonged VR training protocols and activities that closely mimic daily functional tasks to enhance real-world applicability. Additionally, including diverse stroke populations and various rehabilitation settings with a longer follow-up duration in future studies would help validate and expand upon these findings. By refining VR training protocols and ensuring equitable access to this innovative therapy, we can better support stroke survivors’ recovery journey.

## Figures and Tables

**Table 1:** Demographics of participants and baseline outcome indicators.

	All Participants	Experimental Group (EG)	Comparison Group (CG)	t/x^2^	*p*
	*n* = 33	*n* = 16	*n* = 17		
Age (years)	61.5 ± 11.1	59.9 ± 12.8	63.1 ± 9.3	0.676	0.417
Sex (male)	25 (75.8)	13 (81.2)	12 (70.6)	0.51	0.475
Body mass index (kg/m^2^)	25.6 ± 4.7	26.2 ± 4.8	24.9 ± 4.7	0.563	0.459
Education				3.735	0.443
Under elementary school	1 (3.0)	0 (0)	1 (5.9)		
Elementary school	7 (21.2)	4 (25.0)	3 (17.6)		
Junior high school	6 (18.2)	3 (18.8)	3 (17.6)		
Senior high school	7 (21.2)	5 (31.2)	2 (11.8)		
College and above	12 (36.4)	4 (25.0)	8 (47.1)		
Marital status				0.279	0.870
Unmarried	7 (21.2)	4 (25.0)	3 (17.6)		
Married	24 (72.7)	11 (68.8)	13 (76.5)		
Widowed	2 (6.1)	1 (6.2)	1 (5.9)		
Caregiver				6.686	0.153
None	2 (6.1)	2 (12.5)	0 (0)		
Family members	24 (72.7)	12 (75.0)	12 (70.6)		
Foreigner maid	1 (3.0)	1 (6.2)	0 (0)		
Professional caregiver	6 (18.2)	1 (6.2)	5 (29.4)		
Paraside/Affected side (Right)	19 (57.6)	7 (43.8)	12 (70.6)	2.431	0.166
Duration of stay	17.8 ± 6.6	17.8 ± 6.6	17.9 ± 6.8	0.003	0.955
Comorbidities					
Hypertension	23 (69.7)	11 (68.8)	12 (70.6)	0.013	1.000
Type 2 diabetes	7 (21.2)	5 (31.2)	2 (11.8)	1.873	0.225
Heart disease	9 (27.3)	5 (31.2)	4 (23.5)	0.248	0.708
Hyperlipidemia	23 (69.7)	11 (68.8)	12 (70.6)	0.013	1.000
Metabolic syndrome	6 (18.2)	4 (25.0)	2 (11.8)	0.971	0.398
Discharge destination				1.873	0.225
Home	26 (78.8)	11 (68.8)	15 (88.2)		
Post-acute care units/Nursing home	7 (21.2)	5 (31.2)	2 (11.8)		

Note: Data were presented as numbers (%) in categorical variables or mean ± standard deviation (SD) in continuous variables; *p* values were derived from a Student’s *t*-test (continuous variable) or a chi-squared test (categorical variable). Abbreviations: CG, control group; EG, experimental group.

**Table 2:** Differences in outcome indicators between groups at baseline and after the intervention.

	All (*n* = 33)	EG (*n* = 16)			CG (*n* = 17)			Baseline		Post-Intervention	
	Baseline	Baseline	After	*p*	Baseline	After	*p*	EG vs. CG		EG vs. CG	
								*t*	*p*	*t*	*p*
Muscle strength											
Affected side											
Upper extremity	3.5 ± 1.2	3.4 ± 1.3	4.3 ± 1.3	0.082	3.5 ± 1.2	4.1 ± 1.6	0.192	0.01	0.940	0.07	0.794
Lower extremity	3.8 ± 0.8	3.7 ± 0.6	4.6 ± 0.8	0.001	3.8 ± 0.9	4.5 ± 0.9	0.047	0.26	0.611	0.25	0.618
Unaffected side											
Upper extremity	4.7 ± 0.5	4.6 ± 0.6	4.9 ± 0.3	0.034	4.8 ± 0.4	5.0 ± 0.0	0.073	2.07	0.160	1.07	0.310
Lower extremity	4.5 ± 0.8	4.5 ± 0.8	4.9 ± 0.5	0.128	4.5 ± 0.7	4.8 ± 0.4	0.085	0.01	0.913	0.11	0.744
NHISS	1.6 ± 1.0	1.7 ± 1.3	1.9 ± 2.2	0.775	1.5 ± 0.5	1.4 ± 0.5	0.315	0.22	0.646	0.88	0.357
Depression	10.2 ± 4.5	12.1 ± 1.4	9.2 ± 2.2	<0.001	8.5 ± 5.8	9.2 ± 5.6	0.719	5.83	0.022	0.00	0.994
Anxiety	10.9 ± 5.2	12.5 ± 2.4	11.0 ± 2.3	0.090	9.4 ± 6.6	8.9 ± 5.8	0.827	3.20	0.083	1.83	0.186
mRS	3.5 ± 0.9	3.3 ± 0.9	2.7 ± 1.2	0.112	3.7 ± 0.8	2.5 ± 1.1	0.001	1.23	0.277	0.16	0.691
PASS	20.6 ± 12.0	21.0 ± 12.3	30.0 ± 8.0	0.020	20.1 ± 12.1	24.2 ± 12.5	0.336	0.04	0.837	2.45	0.128
FIM	88.2 ± 28.0	95.4 ± 23.5	108.3 ± 17.5	0.088	81.4 ± 30.9	94.9 ± 34.2	0.235	2.11	0.156	1.95	0.173
Barthel Index	46.7 ± 24.2	52.5 ± 23.0	57.2 ± 27.6	0.605	41.2 ± 24.7	53.5 ± 29.5	0.195	1.85	0.183	0.14	0.716

Note: *p* values were from a paired *t*-test, student *t*-test, or chi-squared test. Abbreviations: CG, comparison group; EG, experimental group; FIM, Functional Independence Measure; mRS, modified Rankin Scale; NHISS, National Institute of Health Stroke Scale; PASS, Postural Assessment Scale for Stroke.

**Table 3:** Effect of the intervention on outcome indicators based on generalized estimating equations (GEE) analysis.

	Within Group		Between Group		Interaction Group (EG) × Time				Interaction a Group (EG) × Time			
	Ref: Baseline		CG vs. EG		Reference Group (CG) × Time				Reference Group (CG) × Time			
	*ß*	*p*	*ß*	*p*	*ß*	95% C.I.		*p*	*ß*	95% C.I.		*p*-Adjusted ^a^
						Lower	Upper			Lower	Upper	
Muscle strength												
Affected side												
Upper extremity	0.81	0.063	0.03	0.937	−0.17	−1.42	1.09	0.797	−0.29	−1.12	0.55	0.500
Lower extremity	0.94	<0.001	0.14	0.592	−0.29	−1.05	0.47	0.456	−0.33	−0.88	0.21	0.232
Unaffected side												
Upper extremity	0.38	0.022	0.26	0.143	−0.20	−0.57	0.17	0.291	−0.24	−0.53	0.05	0.107
Lower extremity	0.38	0.106	−0.03	0.910	−0.02	−0.61	0.57	0.942	−0.09	−0.46	0.29	0.642
NHISS	0.19	0.766	−0.16	0.640	−0.36	−1.64	0.91	0.576	−0.47	−1.34	0.39	0.286
Depression	−2.88	<0.001	−3.59	0.01	3.58	−0.32	7.48	0.072	3.77	0.87	6.67	0.011
Anxiety	−1.50	0.070	−3.15	0.059	1.03	−3.35	5.41	0.645	1.03	−1.72	3.78	0.463
mRS	−0.63	0.090	0.34	0.256	−0.49	−1.44	0.45	0.308	−0.50	−1.09	0.10	0.100
PASS	9.00	0.011	−0.88	0.830	−4.88	−15.50	5.74	0.367	−4.62	−11.2	1.93	0.167
FIM	12.9	0.069	−14.0	0.130	0.65	−24.7	26.0	0.960	−0.91	−15.5	13.7	0.903
Barthel Index	4.7	0.590	−11.3	0.159	7.67	−16.9	32.3	0.541	8.52	−10.3	27.3	0.374

Note: Analyses were performed by GEE models, with a group × time interaction term characterizing the intervention effect of interest. ^a^ Models were adjusted for sociodemographic factors and comorbidities (i.e., sex, age, educational level, marital status, body mass index, caregiver, duration of stay, hypertension, type 2 diabetes, heart disease, hyperlipidemia, and metabolic syndrome). Abbreviations: CG, comparison group; EG, experimental group; FIM, Functional Independence Measure; mRS, modified Rankin Scale; NHISS, National Institute of Health Stroke Scale; PASS, Postural Assessment Scale for Stroke; *ß*: Regression coefficient.

## Data Availability

The data used and analyzed during the current study are available from the corresponding author upon reasonable request.
